# Jefferson Medical College

**Published:** 1843-03-04

**Authors:** 


### JEFFERSON MEDICAL COLLEGE.

By the annual catalogue of this Institution for the session 1842–'43, we find the number of students to be two hundred and twenty-nine. At the public commencement on the 10th March, 1842, the degree of Doctor of Medicine was conferred on fifty-nine pupils.

